# Expression of the *zic1*, *zic2*, *zic3*, and *zic4 *genes in early chick embryos

**DOI:** 10.1186/1756-0500-3-167

**Published:** 2010-06-16

**Authors:** Ariel R McMahon, Christa S Merzdorf

**Affiliations:** 1 Department of Cell Biology and Neuroscience, Montana State University, Bozeman, MT 59717, USA

## Abstract

**Background:**

The *zic *genes encode a family of transcription factors with important roles during early development. Since little is known about *zic *gene expression in chick embryos, we have characterized the expression patterns of the *zic1, zic2, zic3, and zic4 (zic1-4) *genes during neurulation and somitogenesis.

**Findings:**

We used *in situ *hybridization to analyze the expression patterns of the *zic1-4 *genes during early chick development (HH stages 7-19). The *zic1-3 *genes showed both overlapping and gene-specific expression patterns along the length of the dorsal neural tube and in the dorsal parts of the somites. In addition, unique expression domains of *zic *genes included: *zic2 *in the neural plate, periotic mesoderm and limb buds; *zic3 *in the paraxial mesoderm surrounding the neural plate, in presomitic mesoderm and in the most recently formed epithelial somites; *zic2 *and *zic3 *in developing eyes. *zic4 *expression was limited to dorsal fore- and midbrain regions and, unlike the expression of the *zic1-3 *genes, *zic4 *expression was not detected in the hindbrain and trunk. This was in contrast to more extensive *zic4 *expression in other vertebrates.

**Conclusions:**

The *zic1-3 *genes were expressed in both overlapping and unique domains within the neural tube, somites and other ectoderm and mesoderm-derived structures in the future head and trunk. *zic4 *expression, however, was limited to dorso-anterior regions of the future brain. This is the first comprehensive study of *zic1-4 *gene expression in chick embryos during neurulation and somitogenesis.

## Background

The *zic *genes encode a family of zinc finger transcription factors. Five *zic *genes are typically found in vertebrates (*zic1-5*), where they guide a variety of developmental processes [1] and *zic *genes play significant roles during early neural patterning and neural crest formation [2-11]. Although *zic *genes may be able to partially compensate for each other [12,13], mutations in individual *Zic *genes in mice and humans produce distinct phenotypes [14]. For example, compromised expression of the *Zic2 *gene results in neural tube defects and mutation of the *Zic3 *gene causes left-right abnormalities in addition to less severe neural tube defects [15-23]. In addition, mutations in the *Zic1 *or *Zic4 *genes result predominantly in cerebellar abnormalities [13,24]. Collectively, these phenotypes illustrate the relevance of *zic *genes to early developmental processes. However, much further study is required to elucidate the mechanisms that underlie the phenotypes associated with mutations of individual *zic *genes.

Chick embryos represent a major developmental model system that remains largely untapped for the study of *zic *genes. This is partly due to a lack of characterization of *zic *gene expression during chick development, which has been mainly restricted to studies of the *zic1 *gene [25-28]. Specifically, there are no published micrographs of *zic2 *gene expression patterns prior to stage 23 [27] and descriptions of *zic3 *expression are limited to brain and anterior trunk regions at stages 10, 12, and 17 [29], and to a stage 26 embryo [28]. In addition, the expression of *zic4 *in chick embryos has not been described. In this study, we thoroughly examine *zic1-4 *gene expression in stage 7-19 chick embryos during neurulation and somitogenesis. Care was taken to use gene-specific probes in order to avoid potential cross reactivity among *zic *genes. This study differs from previous work by examining *zic1-3 *expression at earlier stages of chick development (beginning with Hamburger Hamilton HH stage 6+/7), in reporting for the first time on the expression of the *zic4 *gene, and by providing a comprehensive comparison of *zic1-4 *expression patterns up to stage 18/19. Thus, this is the most thorough comparative expression study of the *zic1-4 *genes during early development in chick embryos. As such, it contributes a foundation for future studies of Zic transcription factors during early neural and somite development, where *zic *genes are known to play important roles.

## Results and Discussion

### Genomic location of the zic1-4 genes

The genomes of most vertebrates contain five *zic *genes (reviewed in Merzdorf, 2007). In the chicken genome, the *zic1 *and *zic4 *genes are adjacent and transcribed in opposite directions on chromosome 9, while the *zic2 *and *zic3 *genes are located on chromosomes 1 and 4, respectively. In other vertebrates, the *zic1 *and *zic4 *genes and the *zic2 *and *zic5 *genes are adjacent and transcribed in opposite directions. However, *zic5 *has not yet been identified in the chicken genome. In mouse, the *Zic5 *gene is located between the *Zic2 *and *Clybl *genes. The equivalent intergenic region in the chicken genome sequence contains several gaps and will thus require additional work to conclusively demonstrate the presence or absence of a *zic5 *gene.

Members of the *zic *family show high sequence similarity, particularly throughout their zinc finger domains and in the regions immediately flanking them. Therefore, we used the published cDNA sequence for chicken *zic1 *[25] and the sequence of the chicken genome to select regions for the *zic1-4 *genes that were highly specific for each gene. Antisense RNAs for *in situ *hybridization were synthesized from these regions and used to study *zic1-4 *gene expression in whole embryos and cryosections.

### Comparison of zic1-4 gene expression patterns

We describe *zic1-4 *gene expression during neurulation and somite formation in chick embryos spanning stage HH 6/7 to stage HH 18/19. Head neural fold formation begins at stage 6/7, somite formation at stage 7, and neural tube closure starts at the level of the mesencephalon at stage 8 (4-6 somites). During stages 6-15, gastrulation, neural plate formation, somite formation, and neural tube closure progress in an anterior to posterior direction. In stage 18/19 embryos, the posterior neuropore has closed, the tailbud has formed and limb buds have begun to develop [30,31]. The expression levels of the *zic1-3 *genes were extremely low and detectable only slightly above background in early embryos at stages 6/7 and up to stage 12. The *zic4 *gene was not expressed in these early embryos. In stage 14/15 and 18/19 embryos, the expression of the *zic1-3 *genes in the head was substantially stronger and the *zic4 *gene was expressed in head regions.

#### Brain

**Figure 1:**
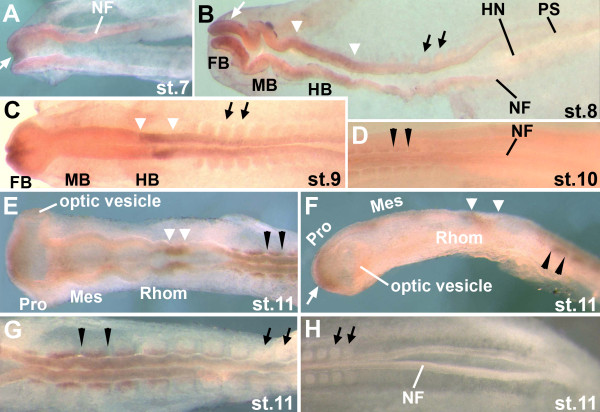
**Expression of the *zic1 *gene in whole mount chick embryos at HH stages 7-11 determined by *in situ *hybridization**. A: In stage 7 embryos, *zic1 *is expressed at very low levels in the most anterior portion of the head fold (white arrow). B: *zic1 *is expressed in the neural folds of the future forebrain (white arrow) and hindbrain (white arrowheads) at stage 8. It is not expressed in the future spinal cord or in somites (black arrows). C: By stage 9, *zic1 *is expressed in the anterior region of the future forebrain and in a domain of the future hindbrain (white arrowheads). D: Very weak *zic1 *expression in anterior somites is first observed at stage 10 (black arrowheads). E (dorsal view), F (side view): *zic1 *expression in anterior somites becomes more robust by stage 11 (black arrowheads), as does *zic1 *expression in the anterior portion of the trunk neural tube (E). In the brain, *zic1 *continues to be expressed in the anterior prosencephalon (best seen in F, white arrow) and in the limited hindbrain domain (best seen in E, white arrowheads). *zic1 *is not expressed in the optic vesicles. G: At stage 11, anterior somites express *zic1*, while it is not expressed in posterior somites. *zic1 *expression in the trunk neural tube diminishes towards posterior regions. H: *zic1 *is not expressed in the neural tube, neural folds, or somites of the posterior trunk in stage 11 (and older) embryos. Overall, expression levels of *zic1 *are very low in these early stage embryos. FB: future forebrain; MB: future midbrain; HB: future hindbrain; HN: Hensen's Node; PS: primitive streak; NF: neural folds; Pro: prosencephalon; Mes: mesencephalon; Rhom: rhombencephalon; white arrowheads: hindbrain expression domain of *zic1*; white arrow: forebrain expression domain of *zic1*; black arrows: somites not expressing *zic1*; black arrowheads flank somites expressing *zic1*; the anterior of the embryos in all figures is to the left.

**Figure 2:**
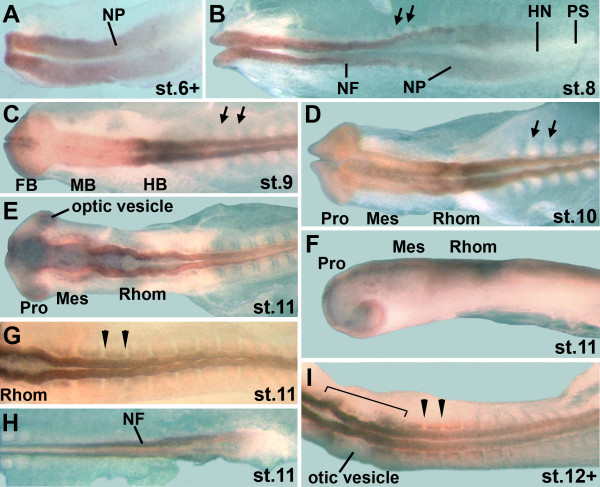
**Expression of the *zic2 *gene in chick embryos at HH stages 6+ to 12**. A: *zic2 *is expressed in all of the head fold and neural plate region of stage 6+ embryos. B: At stage 8, *zic2 *expression continues in the neural folds and throughout the neural plate along the entire anterior-posterior axis of the embryo. C: *zic2 *expression is downregulated in much of the forebrain and midbrain regions of stage 9 embryos. *zic2 *expression persists in a small domain of the forebrain and in a large region of the hindbrain. Contiguous with its expression in the hindbrain, *zic2 *is expressed in the trunk neural tube. D: *zic2 *is not yet expressed in somites of stage 10 embryos. E, F: dorsal view (E) and side view (F) of stage 11 embryo; although *zic2 *expression appears weaker in the mesencephalon region (E), the side view shows that *zic2 *expression is present along the entire dorsal brain and trunk neural tube (F). The optic vesicles express *zic2 *at stage 11. G: Weak *zic2 *expression begins in the anterior somites of stage 11 embryos. H: *zic2 *is expressed in the most posterior neural tube and neural folds. I: in stage 12+ embryos, *zic2 *expression in anterior somites is more robust and *zic2 *is expressed in the periotic mesoderm (bracket). Overall, expression levels of *zic2 *are very low in early stage embryos. Abbreviations as in legend for Figure 1; NP: neural plate; black arrows: somites not expressing *zic2*; black arrowheads flank somites expressing *zic2*; bracket: periotic mesoderm.

**Figure 3:**
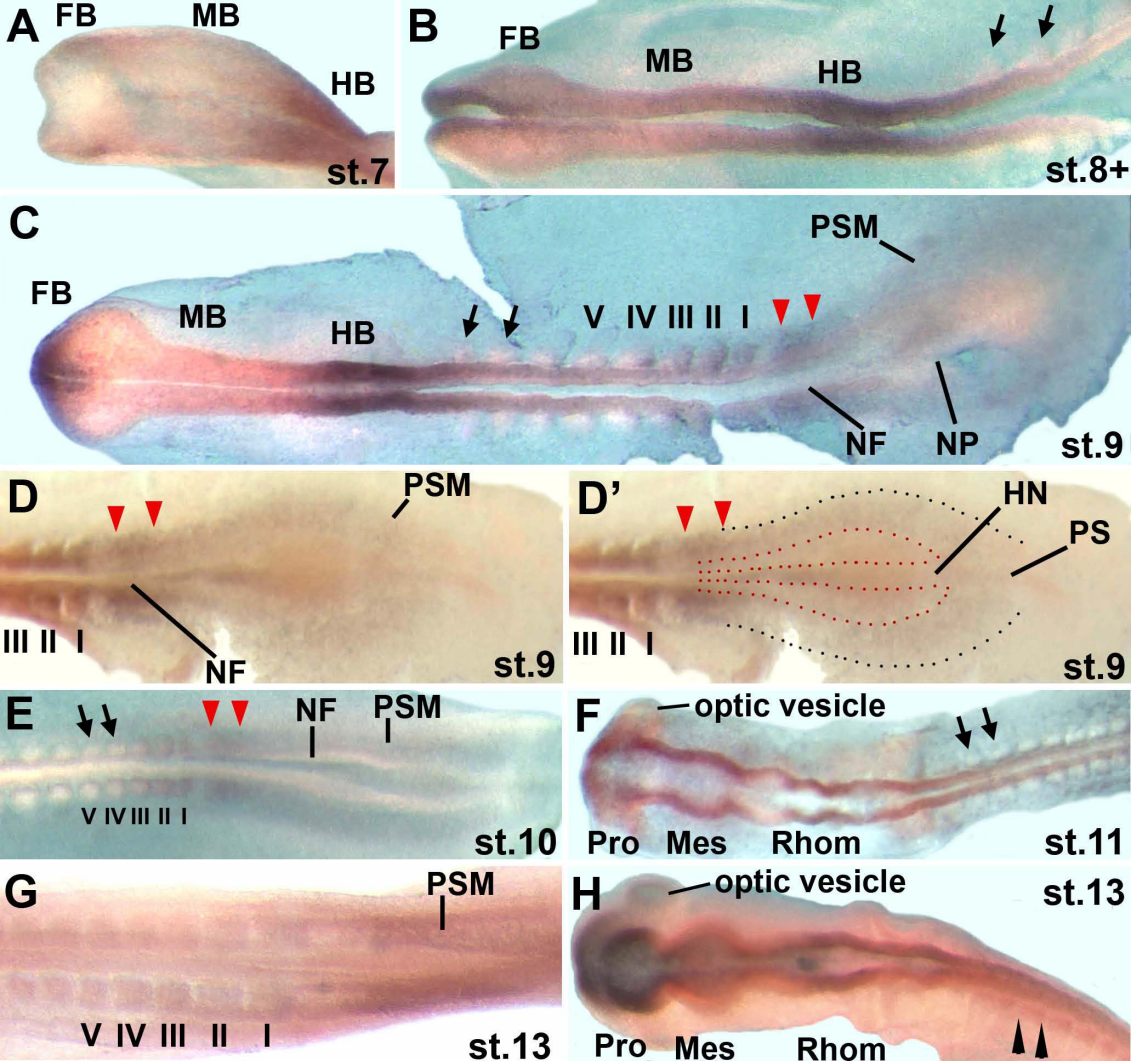
**Expression of the *zic3 *gene in chick embryos at HH stages 7 to 13**. A: *zic3 *is expressed in the future forebrain in a limited and weak expression domain. It is broadly expressed in the future hindbrain. B: At stage 8+, weak *zic3 *expression continues in a limited region of the forebrain. It is expressed in the neural folds of the hindbrain and more weakly in the anterior trunk. C, D, D': In stage 9 embryos (7-9 somites), the first epithelial somites (somites I-III and sometimes somite IV) express *zic3*, while more anterior somites do not express *zic3 *(black arrows, C). *zic3 *is expressed at low levels in the presomitic mesoderm and at a slightly increased level in the area that will form the next somite (red arrowheads). D and D': posterior region of a stage 9 embryo. In D', the area that represents the neural plate and expresses *zic2 *(see Figure 2B) is outlined with red dots. The outer limit of the adjacent presomitic mesoderm, which expresses *zic3*, is marked by black dots. E: At stage 10, low levels of *zic3 *expression continue in presomitic mesoderm and in epithelial somites I-III. The neural folds do not express *zic3*. F: *zic3 *is first expressed along the entire brain in stage 11 embryos. Anterior somites do not express *zic3 *(black arrows). G, H: By stage 13, anterior somites (H, black arrowheads) and the optic vesicles first express low levels of *zic3*. The presomitic mesoderm expresses *zic3*, while there is very little or no expression of *zic3 *in epithelial somites (G). The brain and anterior neural tube express *zic3 *(H), while the posterior neural tube does not (G). Abbreviations as in legend for Figure 1; NP: neural plate; black arrows: somites not expressing *zic3*; black arrowheads flank somite showing weak *zic3 *expression; red arrowheads: increased *zic3 *expression in presomitic mesoderm.

**Figure 4:**
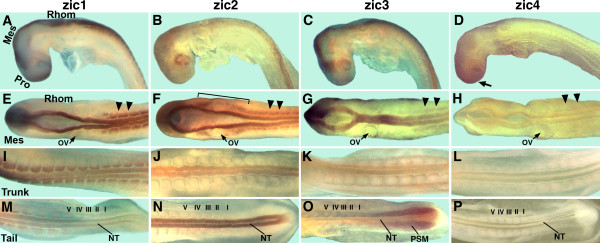
**Comparison of *zic1-4 *gene expression in chick embryos at stage HH 14/15**. A-D: side views of whole embryos; E-H: dorsal views of midbrain, hindbrain, anterior trunk; I-L: mid-trunk; M-P: tail with epithelial somites marked (I - V). A, E, I, M: *zic1 *is expressed in the dorsal brain and in the neural tube, except in the most posterior neural tube (M). *zic1 *is expressed in most somites. B, F, J, N: *zic2 *is expressed along the entire dorsal brain and neural tube of the trunk with enhanced expression in the neural tube of the tail (N). *zic2 *is expressed in the developing eye (B), in the periotic mesoderm (F) and in anterior somites (F). C, G, K, O: *zic3 *is expressed in the dorsal brain, in the eye (C), and in the neural tube, except in the neural tube of the tail (O). *zic3 *is expressed in the presomitic mesoderm (O). D, H, L, P: *zic4 *is expressed in the anterior brain (D) and is not detectable in any other parts of stage 14/15 embryos. Pro: prosencephalon; Mes: mesencephalon; Rhom: rhombencephalon; ov: otic vesicle; NT: neural tube; PSM: presomitic mesoderm; arrowheads outline somites; arrow points to *zic4 *expression; bracket: periotic mesoderm. The stage of the embryos is HH 14 with the exception of D, H: HH 14-; M, F, N, P: HH 15.

**Figure 5:**
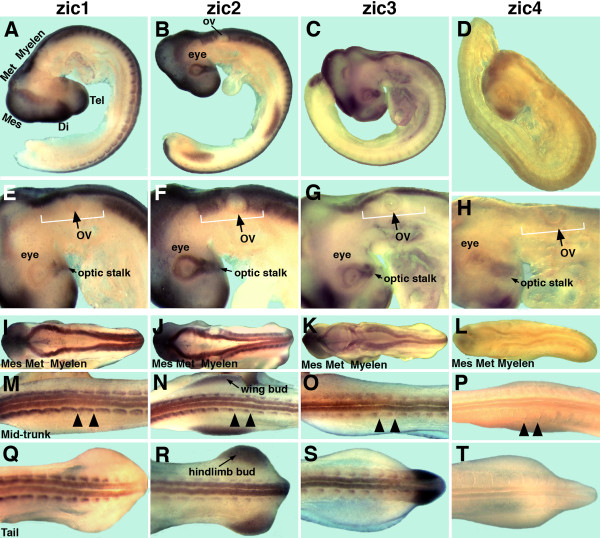
**Comparison of *zic1-4 *gene expression in chick embryos at stage HH 18/19**. A-D: whole embryos; E-H: close-up side-views of the eye and hindbrain regions; I-L: dorsal views of midbrain, hindbrain, and anterior trunk; M-P: dorsal views of the mid-trunk; Q-T: dorsal views of the tail. A, E, I , M, Q: *zic1 *is expressed in the dorsal brain and in the trunk neural tube, except in the tail tip (Q). *zic1 *is strongly expressed in all somites, including caudal somites (Q). B, F, J, N, R: *zic2 *is expressed in the dorsal brain and trunk neural tube. *zic2 *is strongly expressed in the neural tube of the tail tip (B), but more weakly in caudal somites (R). *zic2 *is expressed in the developing eye and periotic mesoderm (B, F) and in the limb buds (B, N, R). C, G, K, O, S: *zic3 *is expressed in the dorsal brain and spinal cord and in the somites. *zic3 *is expressed in the developing eye (C, G) and in the mesoderm of the tail (C, S). D, H, L, P, T: *zic4 *is expressed in the forebrain (D) and midbrain (D, L). No significant expression is detected in the hindbrain or in the trunk. *zic1-4 *are expressed in the optic stalk (E, F, G, H). Tel: telencephalon; Di: diencephalon; Mes: mesencephalon; Met: metencephalon; Myelen: myelencephalon; ov: optic vesicle; white bracket indicates extent of periotic mesoderm; each pair of arrowheads outlines a somite. The stage of the embryos is HH 18 with the exception of A, E, N, R, S, T: HH 19 embryos.

In the developing brain, the expression patterns of *zic1-3 *overlapped extensively, but differed significantly from that of *zic4*. The expression levels of the *zic1-3 *genes were very low in embryos at early stages and became more robust in stage 14 and older embryos. At stage 7, *zic1 *was expressed in the anterior head fold (Figure 1A). In contrast, *zic2 *was expressed in the head fold and along the entire anterior-posterior axis of the neural plate, including the midbrain region (Figure 2A, B). At stage 7, *zic3 *was expressed in discontinuous regions in the neural folds of the future forebrain and hindbrain (Figure 3A). At stage 8, the *zic1 *and *zic3 *genes were expressed in the future forebrain and hindbrain (Figures 1B; 3B). By stage 9, the forebrain expression domains of *zic1-3 *were very similar and well defined (Figures 1C, 2C, 3C). In the midbrain, *zic2 *expression ceased transiently (Figure 2C), resulting in a period during which no *zic *genes were expressed in the midbrain region. In the hindbrain, *zic1 *expression was limited to rhombomere r4 (Figure 1C, E; white arrowheads), while *zic2 *and *zic3 *were expressed in almost identical patterns in most of the hindbrain at stage 9 (Figures 2C, 3C) and throughout the brain at stage 11 (Figures 2E, F; 3F). By stage 14/15, the expression patterns of *zic1-3 *were highly similar and continuous along the forebrain, midbrain, and hindbrain (Figure 4A-C, E-G). Although expression of *zic1-3 *in the brain increased in stage 18/19 embryos, the general expression patterns remained largely unchanged from those at stage 14/15 (Figure 5A-C, I-K). While the expression patterns of *zic1-3 *overlapped extensively, they differed significantly from that of *zic4*. Expression of *zic4 *could only be detected from stage 14, when it was restricted to the anterior telencephalon (arrow, Figure 4D). By stage 18/19, its expression extended to the diencephalon (in a region posterior to that of *zic1-3 *expression) and to the mesencephalon (Figure 5D, H, L), but not to the hindbrain.

The observed expression of the *zic1-3 *genes in the chick forebrain can be related to deficiencies in neural development observed in mice and humans with compromised *Zic *gene expression. Among the *zic *genes in the chick, *zic2 *showed the earliest and most extensive expression in the anterior neural plate/head fold (Figure 2A) and, accordingly, compromised *Zic2 *expression in mice or humans causes holoprosencephaly, in which part of the forebrain does not develop properly [16,20,23,32]. Further, mutations in the *Zic1 *or *Zic3 *genes alone do not cause obvious forebrain abnormalities but *Zic1/Zic3 *compound mutant mice show forebrain defects that are very similar, although not as severe, as those observed in *Zic2 *hypomorphs [11]. This indicates that *Zic1, 2*, and *3 *are involved in forebrain development of mouse embryos. The expression patterns of the *zic1-3 *genes in chick embryos suggest that these genes are involved in forebrain development as well.

The importance of Zic transcription factors in brain development is also suggested by experiments in zebrafish and *Xenopus*. In zebrafish, the *zic2 *gene is required during formation of the anterior diencephalon [33] and the *zic2 *and *zic5 *genes are important during dorsal midbrain development [10]. Further, the *zic1 *and *zic4 *genes are necessary for hindbrain ventricle morphogenesis from the level of r2 towards the posterior [3]. These results corroborate the importance of *zic1 *and *zic2 *during brain development. *Xenopus laevis *embryos express *zic1-5 *during early development [34]. Several genes that are regulated by *zic *genes in *Xenopus *embryos are expressed in domains throughout the brain. For example, Zic1 induces expression of the *wnt1 *and *en-2 *genes and, therefore, is likely to play a role in midbrain/hindbrain boundary (MHB) development [4,5]. Since chicken *zic1 *does not appear to be expressed in the region of the early MHB (Figure 1B), *zic2 *or *zic3 *(Figures 2B, 3B) may potentially play roles in MHB formation in birds. Other genes that are induced by *zic *genes in *Xenopus *include the forebrain/midbrain boundary gene *wnt8b *[5], the dorsal neural tube gene *pax3*, the hindbrain genes *krox20 *[4] and *Xfeb*, which regulates *hoxB1 *expression [35]. *hoxB1 *is expressed specifically in r4 [36], where early chick *zic1 *was expressed (Figure 1C, E). Further, direct targets of Zic1 in *Xenopus *include genes that modulate retinoic acid signaling, suggesting further roles for *zic *genes in hindbrain development [37]. Thus, the expression patterns in the future brain of early chick embryos supports numerous roles for *zic *genes in neural development.

#### Eyes

The developing eyes in stage 9 to stage 18/19 chick embryos did not express *zic1 *or *zic4 *(Figures 1, 4, 5). *zic2 *expression began in the optic vesicles at stage 11 (Figure 2E, F) and *zic3 *expression at stage 13 (Figure 3H). All four *zic *genes were expressed in the optic stalk (Figure 5E-H). The significance of *zic2 *and *zic3 *expression for optic vesicle development remains to be determined.

#### Neural tube of the trunk

In chick, the *zic1-3 *genes were expressed in the neural tube of the trunk. *zic3 *expression preceded *zic1 *expression in the anterior trunk neural tube (stage 7/8 for *zic3*; stage 9 for *zic1*) and, while expression levels were low, the expression of both genes tapered towards the caudal region (Figures 1C; 3B, C). *zic2 *expression in the trunk neural tube was unique, since it was expressed at low levels very early during formation of the neural plate (stage 6+; Figure 2A) and continued to be expressed along the entire trunk neural tube, trunk neural folds and neural plate throughout further early development (Figure 2B-I). Both *zic2 *and *zic3 *were expressed in continuous domains from the hindbrain to the trunk neural tube (Figures 2C, D, G and 3C, F), while *zic1 *expression in the trunk and hindbrain was discontinuous (Figure 1E). As in younger embryos, *zic1 *and *zic3 *expression in the trunk neural tubes of stage 14/15 and stage 18/19 embryos, tapered caudally (in stage18/19 embryos, diminished expression was limited to the tail tips), while *zic2 *expression remained strong to the most posterior extent of the neural folds/neural tube (Figures 4N, R; 5O, S). *zic4 *expression was undetectable in the trunks of embryos at any stage (Figures 4L, P; 5P, T).

**Figure 6:**
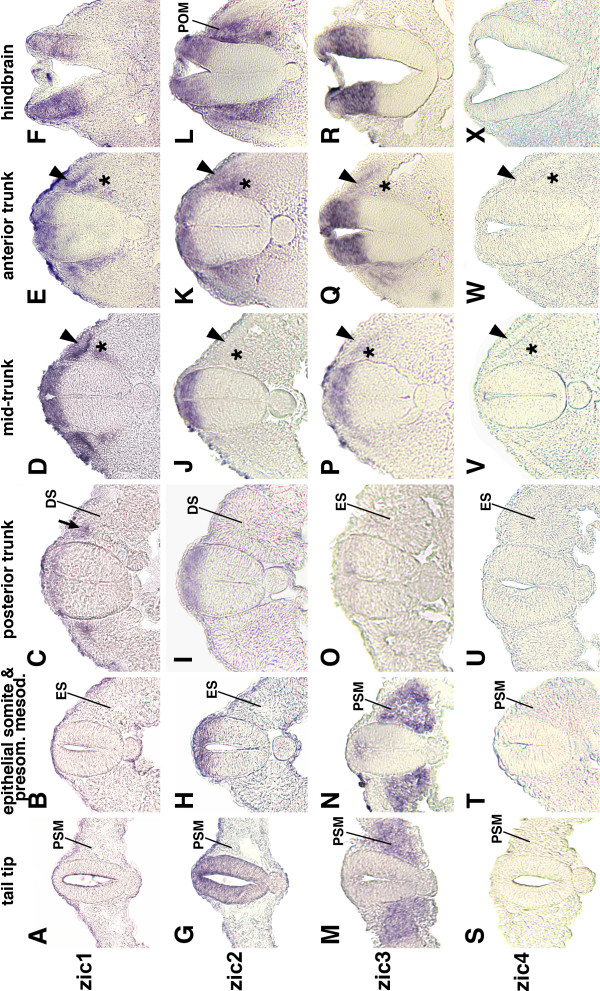
**Sections of trunk and hindbrain regions of stage HH 14/15 embryos**. A-F: *zic1 *expression by *in situ *hybridization. *zic1 *is not expressed in the posterior neural tube (A, B). More rostrally, it is expressed in the dorsal neural tube and in the dorsal portion of recently formed somites (arrow) (C). *zic1 *expression continues in the dorsal neural tube, dermomyotome (arrow heads), and sclerotome (asterisks) (D, E) and in the dorsal hindbrain (F). G-L: *zic2 *expression. *zic2 *is expressed throughout the dorsal/ventral axis of the neural tube in the tail (G). Rostral to the tail region, *zic2 *expression is restricted to the dorsal portion of the trunk neural tube (H-K). *zic2 *is expressed in the dermomyotome and sclerotome of rostral somites (K; arrow head and asterisk, respectively). It is expressed in the dorsal hindbrain and in the periotic mesoderm (L). M-R: *zic3 *expression. *zic3 *is expressed in the presomitic mesoderm (M, N). It is not expressed in epithelial somites (O) and only further rostrally in more mature somites in the sclerotome (P, Q; asterisks) and in the dermomyotome (Q; arrow head). *zic3 *is expressed in the dorsal neural tube of the mid- and anterior trunk (P, Q) and in the dorsal hindbrain (R). S-X: *zic4 *is not expressed in the trunk or hindbrain of stage 14/15 embryos. PSM: presomitic mesoderm; ES: epithelial somite; DS: dissociating somite; POM: periotic mesoderm; arrowheads: dermomyotome; asterisks: sclerotome.

Cross-sections of stage 14/15 embryos allowed more precise location of *zic *gene expression following *in situ *hybridization. The *zic1-3 *genes were expressed in the dorsal neural tubes of the mid-trunk and anterior trunk (Figure 6D, E, J, K, P, Q) and in the dorsal hindbrain (Figure 6F, L, R), whereas *zic4 *was neither expressed in the trunk nor hindbrain (Figure 6S-X). In the tail tip region, *zic2 *was expressed not only in the dorsal portion, but also throughout the remainder of the neural tube (Figure 6G).

In mice, decreased *zic2 *expression causes both holoprosencephaly and spina bifida, a posterior neural tube closure defect [16,20]. Thus, expression of chicken *zic2 *in the neural plate and neural folds (Figure 2B, H) may be required for neural tube closure. This appears to be true for Zic proteins in *Xenopus *and zebrafish, since zebrafish *zic2a *and *zic5 *are necessary for hinge point formation during neurulation [38]. Further, we found that Zic factors directly induce an *aquaporin *gene (*aqp-3b*) in Xenopus that is specifically expressed in the neural folds and may contribute to neural tube closure [37].

#### Somites

During chick somite development, the presomitic mesoderm segments into epithelial somites (somites I-V). As new somites form, older somites compartmentalize into an epithelial dermomyotome and mesenchymal sclerotome. *zic *gene expression in the somites proceeded in two phases. During the first phase only *zic3 *expression occurred in newly formed somites, while in the second phase *zic1-3 *were expressed in more mature somites. Phase 1: In stage 7-10 embryos, *zic3 *was expressed at low levels in presomitic mesoderm, which included a stripe of slightly increased *zic3 *expression, where the next somite pair would form (red arrowheads, Figure 3C, D, D', E). In addition, *zic3 *was expressed in two or three of the most recently formed somites (Figure 3C-E). By stage 12, *zic3 *expression was largely limited to somite I and was uniformly expressed in presomitic mesoderm (not shown). The expression of *zic3 *in the presomitic mesoderm (Figure 3C, D) surrounded the expression domain of *zic2 *in the neural plate (Figure 2B). The relationship between these expression patterns is shown in Figure 3D', where red dots outline the *zic2 *expression domain (neural plate; see Figure 2B) and black dots indicate the outer limit of *zic3 *expression (mesoderm). Phase 2: The *zic1-3 *genes were expressed in the most anterior, mature somites. The onset of very low, but detectable expression in anterior somites was stage 10 for *zic1 *(Figure 1D), stage 11 for *zic2 *(Figure 2G), and stage 12/13 for *zic3 *(Figure 3H). In stage 14/15 embryos, *zic1 *expression extended to the most posterior somites (Figure 4I, M), including the dorsomedial region of recently formed somites (Figure 6C) [26]. *zic2 *and *zic3 *expression, however, was absent from more posterior somites (Figures 4J, K, N, O; 6I, J, O). In stage 14/15 embryos, *zic3 *continued to be expressed in presomitic mesoderm (Figures 4O; 6M, N). Cryosections of mature somites in the anterior trunk showed that *zic1 *and *zic2 *were expressed in both the dermomyotome and in the sclerotome (Figure 6D, E, K) [26], while *zic3 *was expressed in the sclerotome with some expression in the dermomyotome of the anterior trunk (Figure 6P, Q). In stage 18/19 embryos, *zic1-3 *were expressed in somites along most of the anterior/posterior axis (Figure 5M-O, Q-S). Since *zic4 *was not expressed in the trunk, no expression was detected in somites (Figures 4L, P; 5D, P, T; 6U-W). Migrating neural crest cells do not express *zic1 *[26] and serial sections suggest that *zic2 *and most likely *zic3 *are not expressed in migrating neural crest cells (not shown).

In mice, skeletal abnormalities occur when *Zic1, Zic2*, or *Zic3 *expression is compromised. This is presumably due to loss of *Zic *expression in the sclerotome [20,39,40], a region where expression of the *zic1-3 *genes was found in chick embryos (Figure 6D, E, K, P, Q). The ability of Zic proteins to stimulate cell proliferation [10,11,25] may be important for the high rate of proliferation in the dorsomedial lip (DML) of the dermomyotome, where *zic1 *and *zic2 *were expressed in chick embryos (Figure 6D, E, K). In mice, *Zic2 *and *Zic3 *are required for proper somite formation, particularly with respect to early establishment of somitic integrity [11]. It is likely that *zic3 *in chick plays a comparable role due to its similar expression in presomitic mesoderm and epithelial somites. However, the significance of the stripes of *zic *gene expression in the presomitic mesoderm is not known and further analysis will be needed to understand their role in somite formation.

#### Periotic mesoderm

Among the *zic1-4 *genes, *zic2 *was uniquely expressed in the periotic mesoderm. This expression became visible at stage 12 (Figure 2I) and continued in stage 14/15 (whole mount embryo Figure 4F; section Figure 6L) and stage 18/19 embryos (Figure 5F).

#### Limb buds

*zic2 *was expressed in stage 18/19 wing and hindlimb buds (Figure 5B, N, R), while *zic1, 3*, and *4 *transcripts were not detected in limb buds. In a previous study [26], we detected *zic1 *expression in the limb buds of stage 21 embryos. Further studies suggested that *zic1 *expression in limb buds was transient during stages 20/21-24 (unpublished). This is consistent with a lack of *zic1 *expression in the limb buds of stage 26 embryos [28].

### Expression levels of zic genes

The *zic1-3 *genes were expressed at very low levels in chick embryos at early developmental stages (stages 7-11 in particular). Thus, minimal expression levels may be sufficient to mediate Zic function during early neural tube and somite formation. *zic1-3 *expression levels increased significantly with progressing maturity of the cranial region during stages 13-15. Increased *zic1-3 *expression is also seen in the trunk region, although this is not apparent until stages 18-19. This delay may be due to the anterior to posterior gradient of maturity in chick embryos, where cranial regions develop ahead of trunk regions. Higher expression levels of *zic1-3 *during stages 13-15 anteriorly and stage 18-19 in the trunk may be required to help direct cellular decisions relating to proliferation versus differentiation in the maturing cranial and trunk neural tube, and in the maturing somites [25,26,41]. Increased Zic protein levels may impact binding to regulatory sites in Zic target genes and may also affect the stoichiometry with interacting proteins [42-44]. This, in turn, may modulate Zic protein function as a transcriptional activator or repressor and influence the affinities of Zic proteins for specific target sites, leading to context-dependent regulation of target gene sets. That transcript levels are dynamic and can vary at different developmental stages is also illustrated by expression of the *tbx1 *transcription factor gene that is transcribed at very low levels in the cranial region in stage 10 embryos and shows greatly increased expression at stage 12 [45]. Before the significance of the differing *zic *gene expression levels can be truly understood, additional work is needed to identify the relevant transcriptional targets, to correlate *zic *mRNA levels with Zic protein levels, to identify Zic functional partners and potential post-translational modifications, to determine the effect of these factors on Zic protein activity, and to determine their effects on the expression of known target genes.

### Comparison of zic gene expression in different species

The current study in chick embryos indicates that the *zic1-3 *genes are expressed during dorsal axial development, both in the neural tube and in somites, while expression of *zic4 *appears limited to the dorsal brain. In mouse and *Xenopus *embryos, *zic4 *is expressed in the brain and in the trunk and its expression pattern is generally thought to resemble that of *zic1 *at a weaker expression level [34,46,47]. Thus, it was suggested that the adjacent *zic1 *and *zic4 *genes are subject to a certain degree of coordinate regulation. Indeed, in zebrafish, the adjacent *zic2*a and *zic5 *genes possess common regulatory elements [10]. In chick embryos, the expression patterns of the *zic1 *and *zic4 *genes appeared to be quite different, suggesting that coordinate regulation is unlikely for the *zic1 *and *zic4 *genes in birds.

In general, the expression patterns of *zic *genes in the neural tube and somites are relatively similar across species. The similarities of *zic1, zic2*, and *zic3 *gene expression in chick to that in other organisms suggests conserved functions for these genes. However, there are also variations in *zic *gene expression across species. These are particularly evident in the presomitic mesoderm and eyes. *Zic2 *and *Zic3 *are expressed in mouse presomitic mesoderm [11], while only *zic3 *was expressed in chick presomitic mesoderm (this study). Further, *Zic1-3 *are expressed in mouse eyes [47], *zic1 *and *zic2 *in *Xenopus *eyes [34], and *zic2 *and *zic3 *in chick eyes (this study). While these differences may be species-specific, other differences in *zic *gene expression are less consistent. For example, Warner et al. (2003) report *zic1-3 *expression in chick periotic mesoderm, while our findings and a study in mouse showed only *zic2 *expression in periotic mesoderm [46]. Besides species-specific variations, additional explanations for observed differences in *zic *gene expression between species and within the same species may be based on more technical considerations. These may include the possibility of different degrees of probe specificity for individual *zic *genes that were used in previous studies (the probes used in this study were specifically designed to preclude cross-hybridization). Another possible explanation for differences in *zic *gene expression patterns includes the possibility of inaccuracies in the precise staging of embryos within the same species and inaccuracies inherent in comparing equivalent stages across species, which could be very important for transient expression features of particular *zic *genes. Further, it is possible that *zic *genes are alternately spliced, which might cause probes to reveal different expression domains. However, to date, alternate splice forms have not been reported for *zic *genes. Finally, since *zic *genes may be able to compensate for each other, it is possible that the roles of *zic *genes may be distributed slightly differently among *zic *genes in different species, resulting in different expression patterns.

## Conclusions

We have followed the expression of the *zic1-4 *genes during early chick development, resulting in a comprehensive side-by-side study of *zic1-4 *gene expression throughout neurulation and somitogenesis. We find that the *zic1-3 *genes are expressed in partially overlapping domains in the dorsal neural tube and in dorsal portions of somites. In addition, the *zic2 *gene is uniquely expressed along the entire early neural plate and *zic3 *is uniquely expressed in the surrounding presomitic mesoderm, suggesting that Zic2 and Zic3 specifically regulate developmental genes during initial formation of the neural tube and somites, respectively. Further, *zic2 *is expressed in the periotic mesoderm and in limb buds and both *zic2 *and *zic3 *are expressed in developing eyes, suggesting involvement of these genes in regulating the formation of these tissues. We also show that the *zic4 *gene is expressed in dorsal regions of the future head, but does not appear to be expressed in the chick hindbrain or trunk. Overall, *zic *gene expression in chick and other organisms shows significant similarities, indicating that the particular strengths of the chick developmental system will complement current studies of *zic *genes in other organisms. At the same time, the species-specific differences in *zic *gene expression that we observe may point to important evolutionary differences, which are of interest in their own right.

## Methods

### Generation of Gene-Specific Antisense RNA in situ Hybridization Probes

Design of a gene-specific antisense RNA probe for the chicken *zic1 *gene was based on its published sequence [25]. For the chicken *zic2*, *zic3*, and *zic4 *genes, homology to these genes in mouse, human, and *Xenopus *was used to identify exon regions for *zic2*, *zic3*, and *zic4 *in the chicken genome. The identification of homologous regions for chicken *zic2 *and *zic3 *was additionally aided by unpublished partial sequence for these genes, kindly provided by Dr. Sara Ahlgren (Children's Memorial Research Center, Chicago). As expected, we found high homology among the four chicken *zic *genes in the zinc finger region and in the regions flanking the zinc fingers. Comparing the identified sequences, we carefully selected regions that were sufficiently divergent to result in antisense RNA probes that would not cross-react. At the same time, the chosen regions had to be long enough to produce useful probes. Not all regions chosen gave rise to working probes and multiple regions were tested to obtain functional gene-specific probes. For *zic1 *and *zic2*, PCR products were synthesized from stage 18 chick cDNA, which was obtained by reverse transcription of isolated total RNA. These PCR products were TA cloned into pGEM-T (Promega) and transcribed from the resulting plasmids to generate antisense RNA probes. For *zic3 *and *zic4*, T7 promoter-containing PCR products were synthesized from stage 18 chick cDNA. The gel-purified PCR products were used as templates for synthesis of antisense RNA probes using T7 polymerase. The primers used to clone pieces of *zic1 *and *zic2 *and for synthesis of PCR products for *zic3 *and *zic4 *were:

*zic1 *forward: 5'-GCGCTAAAACAAAACAGCGA-3'; *zic1 *reverse: 5'-CTGTATTTACAAGAGGGAGTGGG-3' (497 bp in 3'UTR)

*zic2 *forward: 5'-CCCTCCTCTCCCTCCTCCT-3'; *zic2 *reverse: 5'-ACGCTGATTTCCTCACAACC-3' (441 bp in 3'UTR)

*zic3 *forward: 5'-CAGCAAGGACTCCACGAAAAC-3'; *zic3 *reverse: 5'-C**TAATACGACTCACTATA**GGCGACCCCATCAGATGAGAAT-3' (ca. 730 bp; little 3' coding region and mostly 3'UTR).

*zic4 *forward: 5'-GCTCCAGTTCAAAGCCACAT-3'; *zic4 *reverse: 5'-C**TAATACGACTCACTATA**GGGAGCCAGGTTCACGTTCAG-3' (ca. 600 bp; 5'UTR and 5' coding region). 

The bold bases represent T7 RNA polymerase promoter sequence.

Extensive comparison of the probe sequences that were amplified by these primer sets among each other, comparison of each of these sequences to the regions of all *zic *genes in the chicken genome and to the complete sequences of the *zic2-4 *genes in mouse, human and Xenopus (since we do not have complete sequence information for these three genes in chicken) allowed us to conclude that any cross-reactivity of these probes with other zic genes was extremely unlikely. A lack of cross-reactivity of our probes was further suggested by the gene expression patterns generated by each probe. The expression patterns produced by each of the four probes showed unique features such that none of the staining patterns could be a subset of the staining pattern generated by another one of the probes.

Finally, our exhaustive sequence comparisons of each of the four chicken *zic *genes with all *zic *genes of mouse, human and Xenopus allowed us a very high degree of certainty that we had correctly identified each chicken *zic *gene. Further, our extensive independent comparisons were in agreement with the annotations provided in the Ensembl chicken genome database.

### In situ Hybridization

White Leghorn chicken embryos were staged according to [30]. Whole mount *in situ *hybridization was performed as in [48] with the modifications described in [26]. NBT/BCIP substrate (Sigma) was used for color detection. The embryos were not post-treated with alcohol, since such treatment proved to remove too much of the fainter stain, which was critical for assessing *zic *gene expression domains. Hence the red-brown color of the NBT/BCIP reaction product in our images. A pink shadow was digitally softened in Figure 2F and the orange color in Figure 3H was diminished slightly. Both manipulations did not alter the data content of either panel.

### Cryosections

Embryos were sectioned following *in situ *hybridization. Stained embryos were cryoprotected in graded sucrose solutions and embedded in OCT compound. Cryosections of 14 μm thickness were collected on Superfrost Plus (VWR) slides. The sections were rehydrated in 1× PBS and mounted in 1:1 glycerol/PBS.

## Competing interests

The authors declare that they have no competing interests.

## Authors' contributions

AM performed, analyzed and photographed the *in situ *hybridizations at stages 14/15 and 18/19, cryosectioned and photographed stage 14/15 embryos, and helped edit the manuscript. CM performed *in situ *hybridization and photography of earlier stage embryos, supervised the project, and wrote the manuscript. Both authors read and approved the final manuscript.
